# Monitoring and Evaluation of Corrosion at the Interface of Zirconium Alloy Biomaterials Under Simulated Oxidative Biological Environment

**DOI:** 10.3390/ijms262110537

**Published:** 2025-10-29

**Authors:** Lidia Benea, Veaceslav Neaga, Nicoleta Bogatu, Elena Roxana Axente

**Affiliations:** 1 Competences Centre—Interfaces-Tribocorrosion-Electrochemical Systems (CC-ITES), “Dunărea de Jos” University of Galați, 47 Domneasca Street, 800008 Galați, Romania; veaceslav.neaga@ugal.ro; 2 Interdisciplinary Research Centre in the Field of Eco-Nano Technology and Advance Materials CC-ITI, Faculty of Engineering, “Dunărea de Jos” University of Galați, 47 Domneasca Street, 800008 Galați, Romania; 3 Center for Research and Technology Transfer in the Medico-Pharmaceutical Field, “Dunărea de Jos” University, 800008 Galați, Romania; elena.axente@ugal.ro; 4 Department of Pharmaceutical Sciences, Faculty of Medicine and Pharmacy, “Dunărea de Jos” University, 800008 Galați, Romania

**Keywords:** Zr2.5Nb alloy, electrochemical oxidation treatment, porous oxidized surface, open circuit potential, electrochemical impedance spectroscopy, X-ray diffraction, inflammatory environment

## Abstract

The present work investigates the electrochemical behavior of the Zr2.5Nb alloy in a biomedical context, emphasizing the influence of electrochemical oxidation treatment on its stability in simulated physiological environments. The alloy samples were oxidized in 1 M H_2_SO_4_ under controlled voltages (200–275 V) and times (1 min), identifying 200 V–1 min as the optimal condition for obtaining a uniform porous oxide layer with an average pore diameter of ~90 nm. The corrosion resistance was evaluated using open circuit potential (OCP) and electrochemical impedance spectroscopy (EIS) in Ringer’s solution and Ringer’s solution containing 40 g/L H_2_O_2_ to simulate physiological and inflammatory conditions. Electrochemical tests revealed that electrochemically oxidized samples exhibited a polarization resistance up to 14.78 MΩ·cm^2^, about 26 times higher than that of the untreated alloy (0.56 MΩ·cm^2^). After 77 h of immersion, the oxidized alloy maintained a high resistance (17.54 MΩ·cm^2^), confirming long-term stability. Scanning Electron Microscopy (SEM–EDX) and X-Ray Diffraction (XRD) analyses highlighted significant increases in oxygen content and the transformation from the monoclinic baddeleyite to the cubic arkelite phase of ZrO_2_, contributing to enhanced corrosion resistance. These findings demonstrate that controlled electrochemical oxidation significantly improves the durability of Zr2.5Nb alloy in oxidative environments, supporting its potential for long-term biomedical implant applications.

## 1. Introduction

In the context of biomedical applications, a biomaterial is considered biocompatible when it fulfills the functional and physiological requirements specific to a particular use.

Biocompatibility is therefore context-dependent: a material may be well tolerated and function appropriately in one biological environment or medical scenario but may elicit adverse responses or fail to perform in a different context. For example, a metal alloy may be biocompatible as an orthopedic implant but could be unsuitable for use in cardiovascular devices due to different tissue interactions [1].

A major technological challenge in implant development is the identification and optimization of materials that meet stringent standards of biocompatibility and long-term performance under in vivo conditions. In this direction, recent research is focused on zirconium alloys, which, due to their variable crystal structure and chemical composition, offer promising prospects [2,3,4,5,6].

A notable example is the Zr2.5Nb alloy studied in this work, which, according to the review by Mehjabeen et al. [7], presents multiple biomedical advantages: high biocompatibility, superior corrosion resistance, absence of allergic reactions, increased hardness (~12 GPa), superior aesthetics in dental applications, radiopacity, and good osseointegration. However, it also presents some disadvantages, such as difficulties in intraoral finishing and relatively high costs.

Corrosion of implanted metallic materials is a key issue in assessing the durability and safety of biomaterials. Dissolved salts in body fluids—especially chloride ions (~103 mmol/L in blood plasma)—constitute an aggressive chemical environment, contributing to the acceleration of degradation processes of metallic implants [8,9,10,11]. In addition, electrochemical processes occurring at the metallic surface, particularly partial oxygen reduction reactions (ORRs), can lead to the formation of reactive oxygen species (ROS) such as H_2_O_2_ and HO_2_^−^, which may induce oxidative stress on adjacent tissues [12,13].

Thus, corrosion stability becomes a critical requirement for biomaterials, along with mechanical properties and biocompatibility.

The Zr2.5Nb alloy has been shown to have a modulus of elasticity close to the human cortical bone (30 GPa), adequate mechanical strength, and remarkable fracture resistance. It also exhibits lower magnetic susceptibility and superior chemical stability over time compared to titanium alloys [14,15,16]. To improve performance in biological environments, metallic materials can be subjected to surface modification treatments that target the functional and biochemical properties of the implant. There are three broad categories of methods: (a) mechanical methods, (b) chemical methods, and (c) physical methods [17,18,19]. Among these, anodic oxidation is a well-established electrochemical process for the formation of oxide layers with insulating properties on metallic surfaces, to enhance their subsequent resistance to corrosion and wear in aggressive human intracorporeal environments [20,21,22,23]. It is a cost-effective, simple, and efficient method for the formation of a protective oxide layer, which considerably enhances the corrosion and wear resistance of implants in aggressive intracorporeal environments [20,21,22,23]. Its advantages include low cost, ease of application and precise control of the thickness and morphology of the oxide layer.

However, in the literature, there are still a limited number of studies that directly investigate the electrochemical oxidation of Zr2.5Nb alloy, despite its demonstrated potential for biomedical applications [14,15,16,23,24,25,26]. This gap highlights the need for further experimental research on its electrochemical behavior in simulated physiological environments, to establish optimal strategies for surface protection and functionalization.

The novelty of this study consists of the systematic investigation of the electrochemical oxidation process applied to the Zr2.5Nb alloy, a relatively recent material proposed for biomedical applications, under simulated environmental conditions, both physiological (Ringer’s solution) and inflammatory (Ringer + H_2_O_2_), an aspect rarely addressed in specialized literature. This study provides detailed comparative data on the corrosion behavior of oxidized and non-oxidized surfaces, correlated with morphological, compositional, and structural characterizations, thus contributing to increasing the durability of zirconium-based metallic implants.

## 2. Results and Discussion

### 2.1. Morphological and Compositional Analysis of the Surfaces of the Zr2.5Nb Alloy and the Obtained Oxide Films, Using Scanning Electron Microscopy (SEM–EDX)

#### SEM–EDX Analysis of Untreated and Electrochemically Modified Zr2.5Nb Alloy

**Figure 1:**
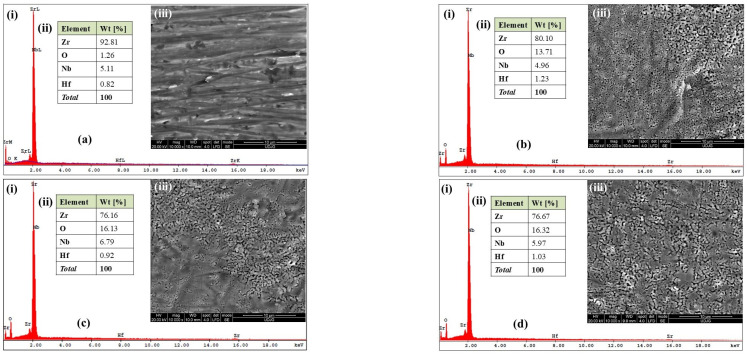
SEM–EDX analysis of (**a**) untreated Zr2.5Nb alloy; (**b**–**d**) electrochemically modified Zr2.5Nb alloy: (**b**) oxidized Zr2.5Nb at 200 V–1 min in H_2_SO_4_; (**c**) Zr2.5Nb alloy oxidized at 250 V–1 min in H_2_SO_4_; (**d**) Zr2.5Nb alloy oxidized at 275 V–1 min in H_2_SO_4_. (i) EDX spectrum of the elements detected; (ii) the quantitative analysis of the detected elements; (iii) micrograph of the studied surface at 10,000× magnification.

Figure 1a shows the SEM–EDX analysis of the untreated Zr2.5Nb alloy. Figure 1(ai) displays the EDX spectrum of the elements detected at a certain ionization energy field (keV), and Figure 1(aii) the quantitative analysis of the detected elements while Figure 1(aiii) shows the micrograph of the studied surface at 10,000× magnification.

According to EDX analysis, the main constituent elements of the untreated Zr2.5Nb alloy in decreasing order of mass percentage values are the following: Zr—92.81%; Nb—5.11%; Hf—0.82%. Another element such as oxygen (O_2_) is identified because the Zr2.5Nb alloy, as well as many other metallic materials, is prone to the formation of thin oxide films after immediate exposure to the ambient environment. In our case, the incorporation percentage of ZrO_2_ on the surface of the untreated Zr2.5Nb alloy is approximately 7.93%.

This result is obtained after converting the mass percentage of oxygen from the general analysis in relation to the molecular mass of zirconium dioxide (123.218 g/mol). In Figure 1(aiii), the surface morphology of the untreated Zr2.5Nb alloy is represented. Thus, the specific rough relief can be observed through unidirectional striations, a configuration resulting from the final processing by the manufacturer.

Figure 1b–d shows the SEM–EDX analysis for Zr2.5Nb alloy samples electrochemically oxidized in 1 mol/L sulfuric acid (H_2_SO_4_) for 1 min at different applied voltages (200 V, 250 V, 275 V). In this electrolyte, the zirconium samples underwent changes; thus, for Zr2.5Nb oxidized at 200 V for 1 min, the mass percentage of ZrO_2_ formation is 54.11%, but in the case of samples electrochemically oxidized at potentials of 250 V and 275 V, a more substantial difference is observed with values of 62.14% and 62.87%, respectively.

Morphologically, on the Zr2.5Nb samples electrochemically oxidized in sulfuric acid, surfaces with different porosities and sizes are highlighted, and in the case of the sample oxidized in 1 M H_2_SO_4_ for 1 min at an applied potential of 275 V, conglomerated pores with larger sizes are observed that unite and form non-uniform crevices (trenches) with random distribution over the entire surface, as shown in Figure 1d.

**Figure 2:**
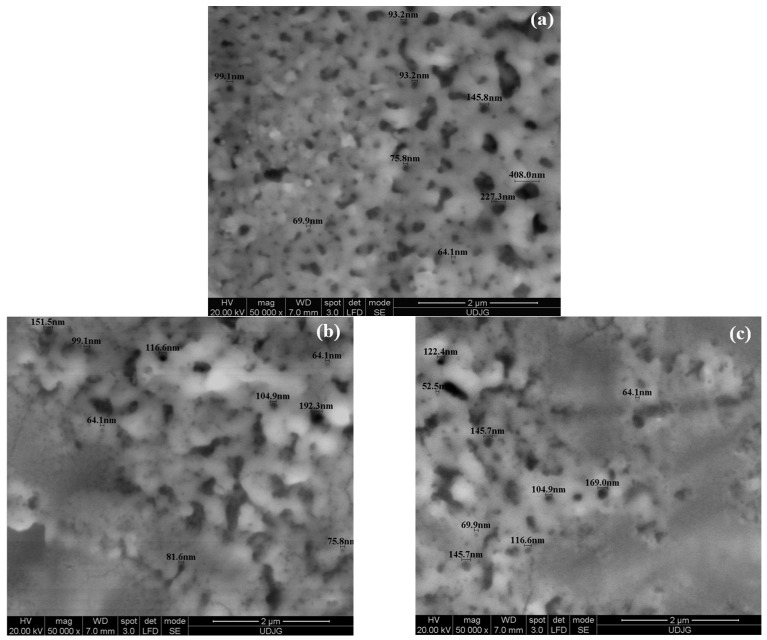
SEM micrographs at 50,000× magnification of electrochemically oxidized Zr2.5Nb samples: (**a**) oxidized Zr2.5Nb (200 V–1 min) in H_2_SO_4_; (**b**) oxidized Zr2.5Nb (250 V–1 min) in H_2_SO_4_; (**c**) oxidized Zr2.5Nb (275 V–1 min) in H_2_SO_4_.

In Figure 2a–c, SEM micrographs of the untreated and electrochemically oxidized Zr2.5Nb alloy are presented for morphological comparison at a higher resolution, a magnitude of 50,000×.

The micrographs in Figure 2a–c represent the surfaces of the Zr2.5Nb alloy after the electrochemical oxidation step in the 1 mol/L sulfuric acid solution, where a non-uniform dissolution of the native zirconium oxide film formed is observed, a phenomenon also encountered in other studies [27], with the presence of a high number of defects and reduced porosity, respectively, decreasing as the applied voltage increases.

From the analysis of Figure 2, a progressive increase in the diameter of the nanopores is observed with the increase in the applied voltage during the electrochemical oxidation process, maintaining the same treatment time (1 min) and the same electrolyte (H_2_SO_4_).

Thus, at a voltage of 200 V, the average value of the pore diameter is 91.58 nm, at a voltage of 250 V, the average value of the pore diameter increases to 124.30 nm while at a voltage of 275 V, the average value of the pore diameter reaches a diameter of 134.50 nm. This evolution indicates that the applied voltage plays an essential role in controlling the size of the nanopores, and increasing its value favors the formation of larger pores.

In addition to the EDX spectral analysis, it is also possible to use the analysis of the distribution maps of the identified elements; thus, in the case of the Zr2.5Nb alloy under study, the elements (O, Zr, Nb, Hf) are analyzed at a magnification of 10,000× of the SEM images.

**Figure 3:**
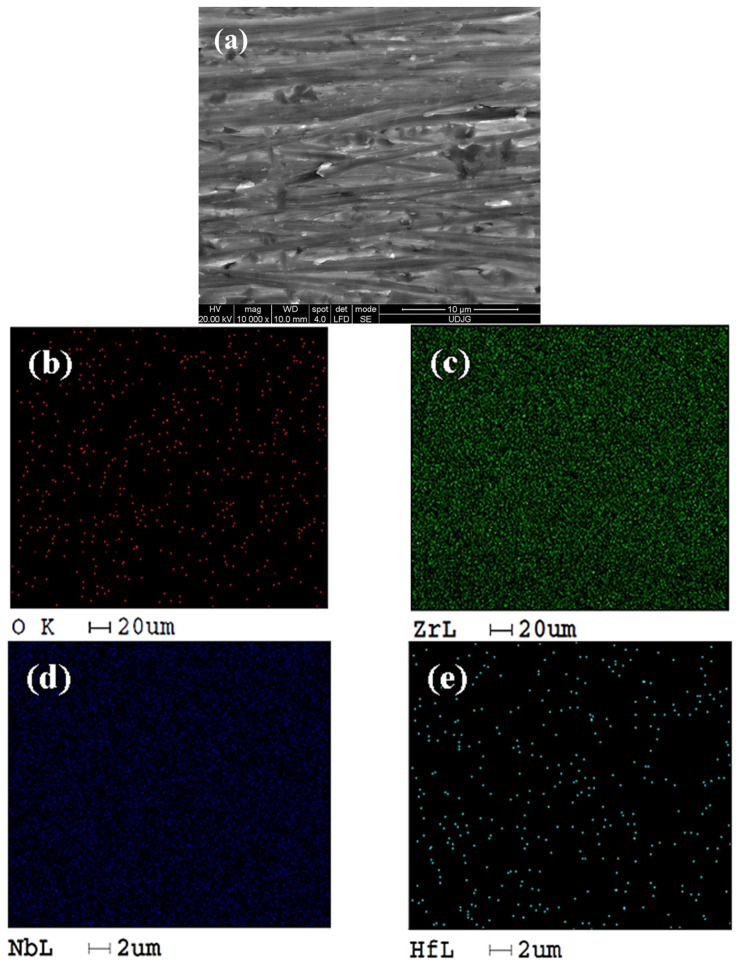
Distribution map of EDX elements identified on the surface study of the untreated Zr2.5Nb alloy at a magnitude of 10,000×: (**a**) SEM image; (**b**) oxygen element distribution; (**c**) Zr element distribution; (**d**) Nb element distribution; (**e**) Hf element distribution.

In Figure 3, the distribution maps of the constituent elements on the surface of the untreated Zr2.5Nb alloy are displayed.

**Figure 4:**
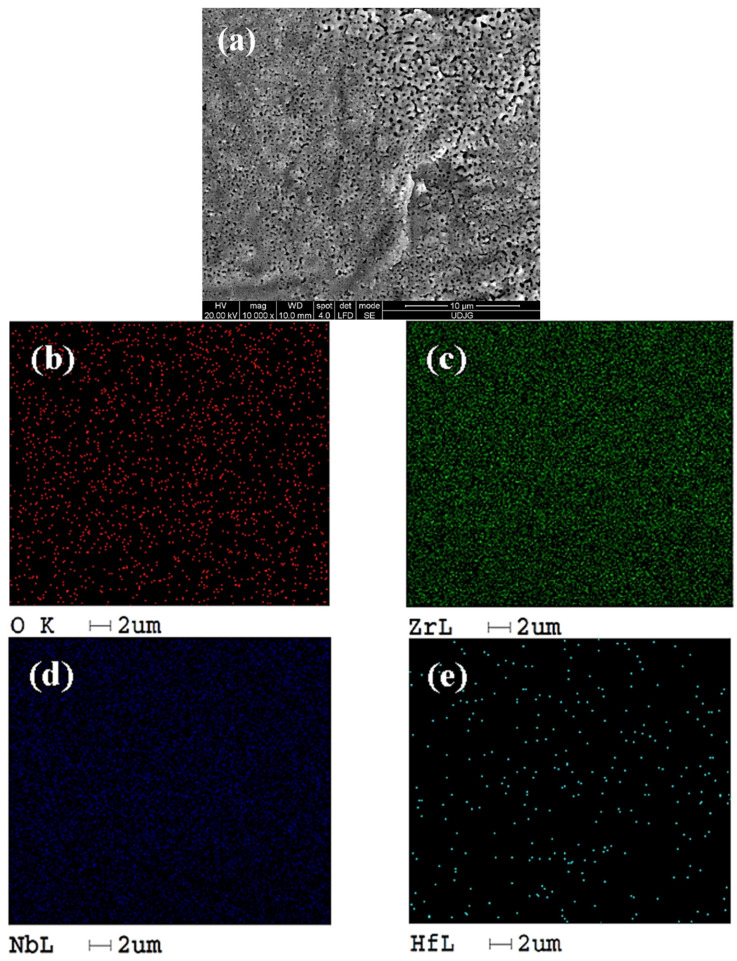
Distribution map of EDX elements identified on the surface study of the oxidized Zr2.5Nb alloy (200 V, 1 min) at a magnitude of 10,000×: (**a**) SEM image; (**b**) oxygen element distribution; (**c**) Zr element distribution; (**d**) Nb element distribution; (**e**) Hf element distribution.

From the analysis of Figure 3, it can be seen that the untreated sample contains less oxygen as compared with the sample oxidized presented in Figure 4b. The spatial distributions of zirconium (Figure 3c), niobium (Figure 3d), and hafnium (Figure 3e) correspond to the expected composition of the alloy. Figure 4 shows the Zr2.5Nb alloy sample anodically oxidized in 1 mol/L sulfuric acid at a voltage of 200 V for a period of 1 min.

From the analysis of Figure 4, it is observed that the presence of oxygen is higher in the anodically oxidized sample, and the other elements are uniformly distributed without obvious changes compared to the untreated sample.

### 2.2. Structural Characterization by X-Ray Diffraction (XRD) of Untreated and Electrochemically Modified Zr2.5Nb Alloy

XRD analysis is based on the ability of the crystalline structures on the surface of a material to diffract X-rays in a characteristic way, which subsequently allows the precise determination of the morphology of the crystalline phases, based on the data obtained. With the help of XRD, the lattice parameters of the chemical element, the space group, the chemical composition, the macro stresses or the qualitative phase analysis can be investigated. Based on the intensity of the recorded peak, information about the crystallographic structure (atomic positions) can be obtained, as well as quantitative phase and texture analyses

**Figure 5:**
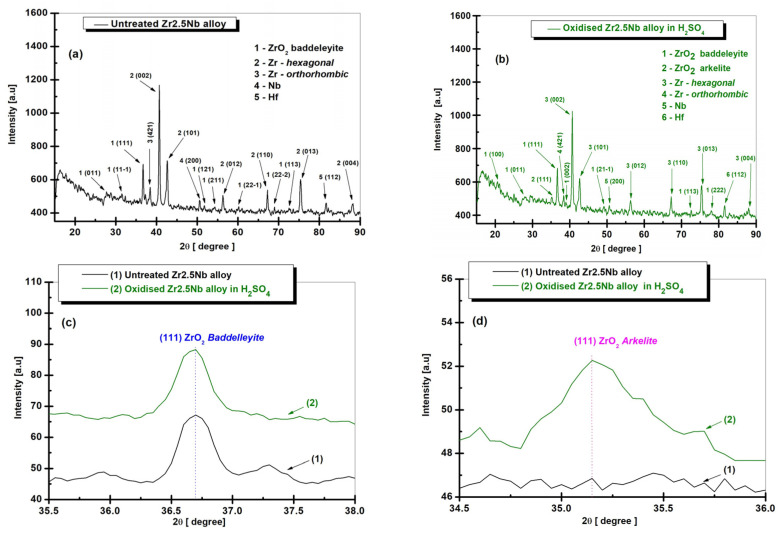
(**a**) XRD spectrum (Co, λKα) with identified crystalline phases for the untreated Zr2.5Nb alloy; (**b**) XRD spectrum (Co, λKα) with the identified crystalline phases for the Zr2.5Nb alloy anodically oxidized in 1M H_2_SO4 at 200 V–1 min. (**c**,**d**) Comparative crystallographic planes of zirconium dioxide obtained after electrochemical oxidation of Zr2.5Nb alloy at 200 V–1 min in H_2_SO_4_.

Finally, the peak shape provides information about the structural phase boundaries of the sample (microstrain variations and crystallite size) [28]. In the field of materials science and engineering, XRD analysis has been developed to become a state-of-the-art technique, especially for qualitative and quantitative phase analysis, but also in investigations of crystallographic textures and residual stress measurements [29]. Figure 5a shows the XRD spectrum for the untreated Zr2.5Nb alloy and Figure 5b shows the XRD spectrum for the electrochemically oxidized and modified Zr2.5Nb alloy.

Thus, for the index noted as (1) in Figure 5a, zirconium dioxide (ZrO_2_) baddeleyite, found in the crystallographic database of the mentioned application with Crystallography Open Database (COD) 96-901-6715, which belongs to the monoclinic crystallization system and the space group P 1 21/c 1, the following crystallographic planes are recognized: (011), (11-1), (111), (121), (211), (22-1), (22-2), and (113) at the 2θ angle levels (28.00°), (32.35°), (36.70°), (51.75°), (54.30°), (60.19°), (69.00°), and (72.59°).

Similarly, after the XRD spectral analysis of the zirconium alloy, the crystalline phase for zirconium (Zr) was identified with the index (2). According to the Crystallography Open Database (COD) 96-900-8524, this phase belongs to the hexagonal crystallization system, space group P63/mmc, with crystallographic planes (002), (101), (012), (110), (013), and (004) corresponding to the 2θ angles (40.68°), (42.64°), (56.32°), (67.21°), (75.35°), and (88.20°). Another type of zirconium identified and mentioned by index (3) is the orthorhombic crystalline form, with space group P b a m, being identified with Crystallography Open Database (COD) 96-231-0735 and the crystallographic plane (421) corresponding to the angle 2θ (38.32°).

In the case of the crystalline phase identified as niobium (Nb), index (4) in Figure 5a, only one crystallographic plane (200) was quantified at the 2θ angle (50.60°), according to Crystallography Open Database (COD) 96-153-4904 with the cubic crystallization system and space group F m −3 m. For the last identified phase—hafnium (Hf), denoted by (5) in Figure 5a—according to the database with COD 96-153-9054, the hexagonal crystallization system, and space group P63/mmc, only one crystallographic plane (112) is observed at the 2θ angle (81.65°).

It is worth noting that the crystallographic planes (002), (101), (012), (110), (013), and (004) for (Zr) zirconium, (111), (11-1), and (211) for zirconium dioxide (ZrO_2_), and (200) with the β phase for niobium (Nb) are also mentioned in other experimental studies with the Zr2.5Nb alloy [30,31,32,33,34,35]. After analyzing Figure 5a, it can be observed that the untreated study sample contains a major Zr phase in a hexagonal structural system, with broader diffraction peaks compared to the position of the oxide diffraction lines at smaller 2θ angles, which indicates the possibility of intercalation of Nb and Hf atoms in the zirconium crystal lattice. This form of crystallographic organization is encountered in a study comparing the Zr2.5Nb alloy with ZrTa and ZrNbTa samples [34,35].

For the Zr2.5Nb alloy anodically oxidized at 200 V–1 min in sulfuric acid (Figure 5b), the difference from the untreated alloy is observed by the additional appearance of crystallographic planes (002), (21-1), and (222)) at the 2θ angles (38.88°), (49.19°), and (78.05°), and the absence of planes (11-1), (121), (211), (22-1), and (22-2) for the baddeleyite-type zirconium dioxide (ZrO_2_) phase. Concomitantly with this modification, another excess crystalline phase (3) is identified, such as zirconium dioxide (ZrO_2_) of the arkelitic type for only a single crystallographic plane (111) at the 2θ angle (35.20°), registered in the database under (COD) 96-500-0039, with the cubic crystallization system in a space group of F m -3 m.

In order to highlight the difference in electrochemical oxidation processes, the sequential comparison of XRD spectra was used according to the most representative crystallographic planes in terms of the intensities of the evolutionary peaks.

According to Figure 5a–d, the untreated Zr2.5Nb alloy samples and the one electrochemically oxidized at a potential of 200 V–1 min are compared at the level of the crystallographic plane (111) of baddeleyite-type zirconium dioxide (ZrO_2_) and according to the sequence of the plane (111) of arkelite-type (ZrO_2_).

Another tool used in XRD structural characterization is the objective differentiation of the average size of ZrO_2_ crystallites, depending on the electrolyte used for anodic oxidation. The evaluation of the average sizes was performed using the Debye–Sherrer Equation (1) [36]:

(1) $$D=\frac{k\lambda }{FWHM*\cos\theta }$$

where *D* is the average crystallite size (Å); *k* is the form factor (0.9); *λ* is the wavelength of the X-ray radiation (1.790300 Å) Co Kα; *θ* is the Bragg angle (or diffraction angle); *FWHM* is the full width at half maximum of the characteristic peak of the peak (in Radians).

Applying this formula and considering that the height corresponds to the average crystallite dimensions calculated according to the (111) plane of ZrO_2_ baddeleyite Figure 5c, the grain size has a value of 49.30 nm for the untreated sample. The size of the oxide grains increases to 50.21 nm in the case of the Zr2.5Nb sample oxidized in sulfuric acid.

In the case of zirconium dioxide (ZrO_2_) arkelite at the (111) crystallization phase according to Figure 5d, the untreated sample does not identify the mentioned plane, and the average crystallite size increases to 40.09 nm for the sample anodically oxidized in H_2_SO_4_.

### 2.3. Corrosion Resistance Assessment of Untreated and Electrochemically Modified Zr2.5Nb Alloy in Both Physiological and Pathological Artificial Solutions

#### 2.3.1. Evolution of the Open Circuit Potential (OCP) for Untreated and Electrochemically Modified Zr2.5Nb Alloy After Immersion in Physiological and Pathological Environments

**Figure 6:**
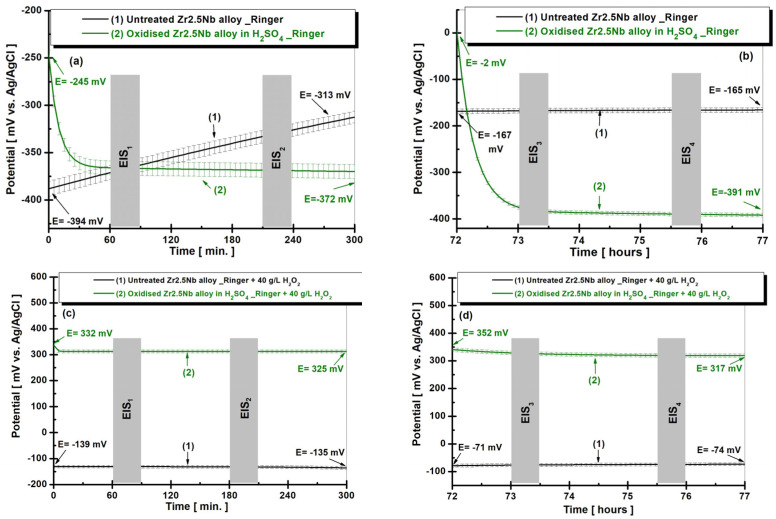
Evolution of the open circuit potential (OCP) for the Zr2.5Nb sample, both untreated (1) and electrochemically oxidized in sulfuric acid (2), after immersion in Ringer’s solution (**a**,**b**), and after immersion in artificial Ringer’s solution with 40 g/L H_2_O_2_ (**c**,**d**) in two time steps: (**a**) t_1_—5 h and (**b**) t_2_—77 h.

The electrochemical behavior of the zirconium alloy (Zr2.5Nb) is evaluated by measuring the evolution of the open circuit potential (OCP) over time, under two different surface treatment conditions and subsequent immersion in the physiological Ringer’s solution. The OCP method is essential in evaluating the stability and reactivity of metallic surfaces in various corrosive environments. It allows for real-time monitoring of the open circuit potential of a material without applying an external current, thus providing valuable information about the processes of formation and dissolution of the oxide layer on its surface. Figure 6 shows the evolution of the open circuit potential for the Zr2.5Nb sample, both untreated and electrochemically oxidized in sulfuric acid, after immersion in the artificial Ringer’s solution, in two time steps: (a) t_1_—5 h and (b) t_2_—77 h.

From Figure 6a, it is observed that the first 300 min of exposure of the samples provided clear insights into the behavior of the untreated and oxidized alloy. The untreated Zr2.5Nb sample (curve 1) shows an increase in the OCP value in the first 300 min of monitoring without reaching the equilibrium state. This sample starts from a potential of −394 mV vs. Ag/AgCl at immersion and reaches a potential of −313 mV vs. Ag/AgCl at the end of the measurement. The potential difference between the value of the potential at immersion and the value at the end of the 300 min in this case is ΔE = 81 mV vs. Ag/AgCl.

The potential shift towards more positive values indicates a rapid formation of the natively formed protective oxide layer on the surface of the material, but as quickly as it forms, it dissolves [37,38,39]. This behavior is also observed in the literature and by other authors [40]. Maciej Sowa and his team [41,42] argue that the presence of a thin native oxide film on the surface of the Zr2.5Nb alloy, not subjected to electrochemical treatment, determines an increase in the open circuit potential due to the reduced permeability of the oxide layer for the corrosive environment, while the slight decrease in the potential observed in the samples treated by electrochemical oxidation can be attributed to the superficial cracks in the porous structure of the formed oxide layer. N. Veaceslav and collaborators [43] also observed a shift of the open circuit potential towards more positive values in the case of untreated Zr2.5Nb alloy

The sample treated with sulfuric acid (curve 2) showed a distinct evolution, with an OCP that is stabilized after approximately 30 min around the value of −372 mV vs. Ag/AgCl. The stability of the open circuit potential suggests the formation of a very stable oxide layer, capable of preventing dissolution and conferring immunity to the corrosive environment [37,38,39]. This constancy indicates that, in this case, the electrochemical oxidation treatment in H_2_SO_4_ solution is more effective in providing protection against corrosion compared to the untreated sample.

The OCP method has demonstrated its utility in evaluating the electrochemical behavior of untreated and electrochemically oxidized zirconium alloys in corrosive environments. By continuously monitoring the open circuit potential, it was highlighted how different surface treatments influence the stability of the oxide layer. In particular, a decrease in OCP indicates the dissolution of the protective oxide layer, while an increase suggests its reformation. A constant OCP signifies the achievement of an equilibrium or immunity state, where the oxide layer remains intact and effective in protecting the material. These results provide valuable insights for optimizing the surface treatments of Zr2.5Nb alloys, in order to improve their corrosion resistance in medical applications.

From Figure 6b, after 3 days of immersion, the samples showed a similar behavior to that shown in Figure 6a. The untreated sample and the one electrochemically oxidized with H_2_SO_4_ maintained their OCPs relatively constant during the tests. This behavior suggests that the formed oxide layer neither dissolves nor forms.

Figure 6c,d shows the evolution of the open circuit potential (OCP) for the Zr2.5Nb sample, both untreated and electrochemically oxidized in sulfuric acid, after immersion in artificial Ringer’s solution with 40 g/L H_2_O_2_ in two time steps: (a) t_1_—5 h and (b) t_2_—77 h.

Comparing Figure 6a–d, it can be seen that samples immersed in Ringer’s solution with the addition of 40 g/L H_2_O_2_ showed more positive potential values compared to those immersed in Ringer’s solution without hydrogen peroxide.

The addition of hydrogen peroxide to Ringer’s solution plays an important role, since H_2_O_2_ is a strong oxidizing agent, which accelerates redox processes on the surface of metallic materials. In this context, H_2_O_2_ can induce the formation of a passivating layer on the alloy surface, providing additional protection against corrosion. In addition, solutions containing H_2_O_2_ are often used to simulate more aggressive conditions, relevant for biomedical applications, such as inflamed body environments or those exposed to oxidative stress. Thus, the study of the interaction of alloys with H_2_O_2_ is essential to evaluate their durability and safety in medical implants or other similar applications [37,38,39,44,45,46,47]. This behavior has also been observed in the literature and by other authors on different materials [37,39,44,45].

#### 2.3.2. Electrochemical Impedance Spectroscopy (EIS) of Untreated and Electrochemically Modified Zr2.5Nb Alloy After Immersion in Physiological and Pathological Environment

Electrochemical impedance spectroscopy is an electrochemical technique based on the application of an alternating current to characterize the processes occurring at the interface of the material (electrode) and the corrosive medium (electrolyte). This method provides a wide range of information about the kinetics of the processes occurring at the electrode–electrolyte interface and is used in various fields, such as the study of corrosion processes, the characterization of semiconductors, the analysis of batteries, and the study of the kinetics of electrochemical deposition [37]. In studies of reactivity and corrosion of metallic surfaces, electrochemical impedance spectroscopy (EIS) plots provide complete information about the kinetics of complex processes or reactions occurring at the electrode–electrolyte interface (e.g., the corrosive environment in which the material under study is immersed) [37].

The EIS plots are presented both in the complex (Nyquist) plane, where the real part is represented on the abscissa and the imaginary part is represented on the ordinate, and in the (Bode) plane with double graphical illustration, where the logarithmic frequency part is represented on the abscissa and the logarithmic impedance modulus or phase angle is represented on the ordinate. The complex representation is often used in the literature because it allows for easy identification of the equivalent circuit elements that are used to fit the recorded experimental data and to determine the polarization resistance or specific resistance [37].

**Figure 7:**
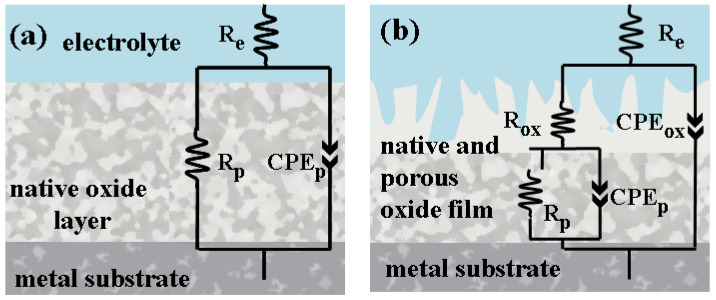
Equivalent electrical circuit used to fit EIS diagrams after corrosion in the electrolytes under study: (**a**) equivalent electrical circuit for untreated samples; (**b**) equivalent electrical circuit for Zr2.5Nb oxidized in sulfuric acid.

Thus, the Nyquist and Bode plots for untreated and electrochemically oxidized Zr2.5Nb samples were fitted using the equivalent electrical circuits shown in Figure 7.

Where R_e_ is the electrolyte resistance, R_p_ is the polarization resistance, CPE_p_ represents the constant phase element of the surfaces of the biomaterials under study, R_ox_ is the resistance of the electrochemically developed oxide, and CPE_ox_ is the constant phase of the electrochemically developed oxide [48,49,50].

**Figure 8:**
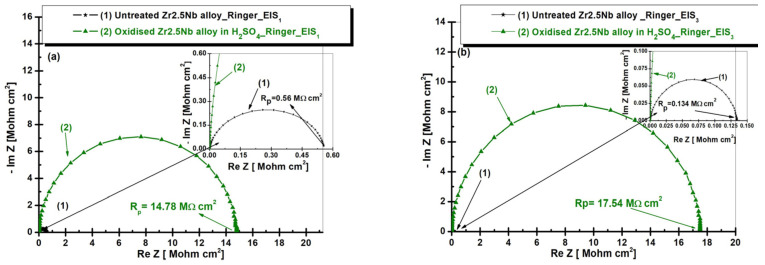
Evolution of electrochemical impedance spectroscopy (EIS) in Nyquist representation for both untreated and electrochemically oxidized Zr2.5Nb samples after immersion in Ringer’s solution. Measurement performed in two time steps: (**a**) t_1_—5 h and (**b**) t_2_—77 h.

Figure 8 shows the EIS evolution in Nyquist representation for both untreated and electrochemically oxidized Zr2.5Nb samples after immersion in artificial Ringer’s solution. The measurement was performed in two time steps: (a) t_1_—5 h and (b) t_2_—77 h.

From Figure 8a, it is observed that after 5 h of immersion, the untreated zirconium alloy (curve 1) has a polarization resistance value approximately 26 times lower compared to the electrochemically oxidized sample (curve 2). The untreated alloy, Zr2.5Nb, has a polarization resistance (R_p_) of 0.56 MΩ·cm^2^, a relatively low value, indicating a reduced protection against corrosion. This suggests that the untreated alloy does not form an effective protective oxide film and is more prone to electrochemical attack.

The Zr2.5Nb sample electrochemically oxidized in sulfuric acid (H_2_SO_4_) solution (Figure 8a, curve (2)) had the highest polarization resistance value, 14.78 MΩ·cm^2^. This treatment produced an extremely efficient protective oxide film, offering superior corrosion protection compared to the untreated alloy. This increase in polarization resistance indicates better passivation and more efficient formation of a protective layer on the alloy surface, thus reducing the corrosion rate.

This phenomenon can be explained by the fact that sulfuric acid favors the formation of a denser and more adherent oxide layer, capable of limiting the transport of ions and electrons compared to the native oxide layer formed on the surface of the untreated alloy [47].

After 77 h of immersion (Figure 8b), significant variations in polarization resistance (R_p_) are observed, which reflect changes in the corrosion behavior of the material, depending on the treatment applied.

From Figure 8b, it is observed that the polarization resistance for the untreated sample (R_p_ = 0.134 MΩ·cm^2^) is significantly lower than for the electrochemically modified sample, indicating a low protection against corrosion. This suggests that the untreated alloy does not form an efficient protective oxide film, which makes it more vulnerable to electrochemical attack in the tested environment (Ringer’s solution). In the case of the Zr2.5Nb sample oxidized in sulfuric acid (H_2_SO_4_) solution, the polarization resistance is the highest, reaching a value of 17.54 MΩ·cm^2^. This denotes the formation of an extremely efficient oxide film, with much better protection against corrosion compared to the untreated alloy. Also, comparing the two immersion times studied, it can be stated that with increasing immersion time, the value of polarization resistance increases for both studied samples. However, the electrochemical treatment applied to the Zr2.5Nb alloy proved to be more effective, demonstrating significantly better protection than the unoxidized samples.

**Figure 9:**
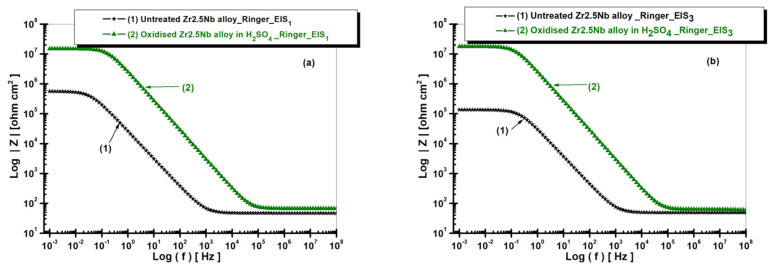
Comparative diagrams of electrochemical impedance spectroscopy (EIS) in Bode representation with the presentation of the impedance modulus as a function of the logarithm of frequency, for both untreated and electrochemically oxidized Zr2.5Nb samples after immersion in Ringer’s solution. Measurement performed in two time steps: (**a**) t_1_—5 h and (**b**) t_2_—77 h.

Figure 9 shows the electrochemical behavior of the Zr2.5Nb alloy in Ringer’s solution, highlighting the significant differences in corrosion resistance depending on the treatment applied. The untreated alloy (curve 1) presents the lowest impedance over the entire frequency range, indicating inadequate passivation and low corrosion protection. This behavior suggests that the protective layer formed under natural conditions is insufficient to limit the corrosive electrochemical processes.

The highest impedance value is recorded for the sample electrochemically oxidized in sulfuric acid solution (curve 2), indicating optimal passivation and maximum corrosion protection. The oxide layer formed in this environment appears to be denser and more adherent, which significantly limits charge transfer and corrosive processes. These results emphasize the critical influence of the oxidizing environment on the corrosion resistance properties of the Zr2.5Nb alloy, demonstrating that oxidation in sulfuric acid solution generates superior electrochemical protection.

**Figure 10:**
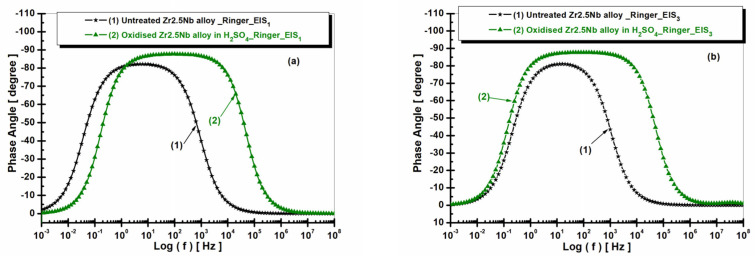
Comparative diagrams of electrochemical impedance spectroscopy (EIS) in Bode format with the representation of the phase angle as a function of the logarithm of frequency, for both untreated and electrochemically oxidized Zr2.5Nb samples after immersion in Ringer’s solution. Measurement performed in two time steps: (**a**) t_1_—5 h and (**b**) t_2_—77 h.

The electrochemical analysis of the Zr2.5Nb alloy in Ringer’s solution is also performed using Bode diagrams, which show the variation of the phase angle as a function of frequency (Figure 10), for two distinct experimental conditions: the untreated alloy and the electrochemically oxidized alloy in sulfuric acid solution (H_2_SO_4_). These measurements allowed for the evaluation of the electrochemical behavior and corrosion protection of the material depending on the treatment applied. In the case of the untreated alloy (curve 1), the phase angle reaches a maximum value of approximately −70°. This value suggests relatively low protection of the alloy against corrosion, which indicates a less stable passivating film and less effective in preventing electrochemical degradation processes. The results obtained for the untreated sample reflect the active electrochemical behavior of the material, without significant protection offered by a passivating layer.

In the case of the alloy electrochemically oxidized in sulfuric acid solution (curve 2), the phase angle reaches a maximum value of approximately −85°, the highest value observed among the analyzed samples. This suggests the formation of an extremely stable and efficient oxide film, which offers the best protection against corrosion among the tested treatments. The result indicates that the electrochemical treatment in sulfuric acid generates a superior quality oxide layer, capable of significantly reducing the rate of corrosion processes. The analysis of the Bode diagram highlights an obvious trend in terms of improving the electrochemical protection of the Zr2.5Nb alloy depending on the treatments applied. Thus, the measured phase angle values suggest that the electrochemical oxidation treatments lead to an increase in the stability of the oxide film and, implicitly, to a more efficient protection against corrosion.

In contrast, the untreated alloy presented the lowest phase angle value, reflecting inadequate corrosion protection. These results highlight the importance of electrochemical treatment conditions in optimizing the corrosion protection performance of zirconium alloys for use as biomaterials and other industrial applications.

Table 1 presents the determined values of the representative equivalent electrical circuit for each type of surface analyzed, after experimental fitting of both untreated and electrochemically oxidized Zr2.5Nb samples in 1 M H2SO4 after immersion in Ringer’s solution.

From Table 1, it can be observed that the evolution of the α parameter over time (EIS_1_/EIS_3_) highlights the changes in uniformity and dielectric behavior of the passive film formed on the Zr2.5Nb alloy surface. For the untreated sample, the α values remain relatively low and nearly constant, indicating a heterogeneous surface characterized by a non-uniform distribution of defects and increased roughness. This behavior is typical of a thin and discontinuous natural oxide film, whose stability changes only slightly during immersion [38].

In the case of the sample electrochemically oxidized in 1 M H_2_SO_4_, the α values are higher compared to the untreated surface and remain close between EIS_1_ and EIS_3_, suggesting a behavior closer to that of an ideal capacitor. This indicates the formation of a dense, homogeneous, and time-stable passive layer with a uniform distribution of dielectric properties. The increase in α after oxidation can be associated with a reduction in surface roughness and an improvement in film continuity, leading to enhanced electrochemical protection [38].

The results obtained from the equivalent electrical circuit analysis demonstrate that the electrochemical treatments applied to the Zr2.5Nb alloy significantly influence its electrochemical behavior. The sulfuric acid treatment led to a significant increase in corrosion resistance and the formation of a more stable oxide film, while the untreated sample showed a more active electrochemical behavior, with a lower corrosion protection, compared to the electrochemically treated samples.

**Figure 11:**
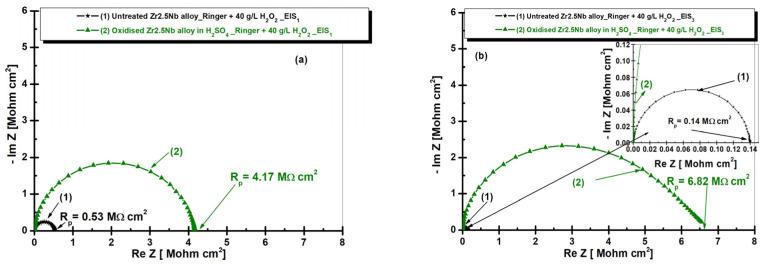
Comparative diagrams of electrochemical impedance spectroscopy (EIS) in Nyquist representation for both untreated and electrochemically oxidized Zr2.5Nb samples after immersion in artificial Ringer’s solution with 40 g/L H_2_O_2_. Measurement performed in two time steps: (**a**) t_1_—5 h and (**b**) t_2_—77 h.

Figure 11 presents the comparative diagrams of the electrochemical impedance spectroscopy (EIS) in Nyquist representation for both untreated and electrochemically oxidized Zr2.5Nb samples after immersion in artificial Ringer’s solution with 40 g/L H_2_O_2_, to simulate infection environment. The measurements are also performed for the two times studied.

Comparing Figure 8 and Figure 11, it can be seen that samples immersed in Ringer’s solution with the addition of 40 g/L H_2_O_2_ showed lower R_p_ values compared to those immersed in Ringer’s solution without hydrogen peroxide, for both times studied.

**Figure 12:**
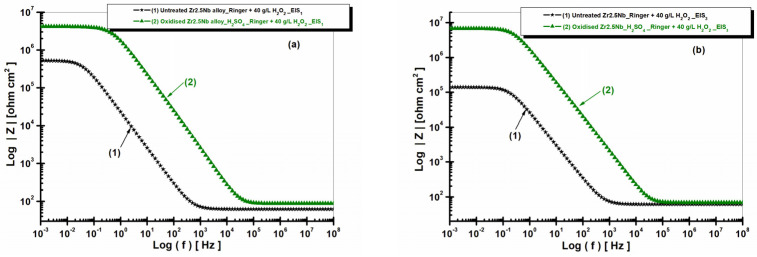
Comparative diagrams of electrochemical impedance spectroscopy (EIS) in Bode format with the representation of the impedance modulus as a function of the logarithm of frequency, for both untreated and electrochemically oxidized Zr2.5Nb samples after immersion in artificial Ringer’s solution with 40 g/L H_2_O_2_. Measurement performed in two time steps: (**a**) t_1_—5 h and (**b**) t_2_—77 h.

**Figure 13:**
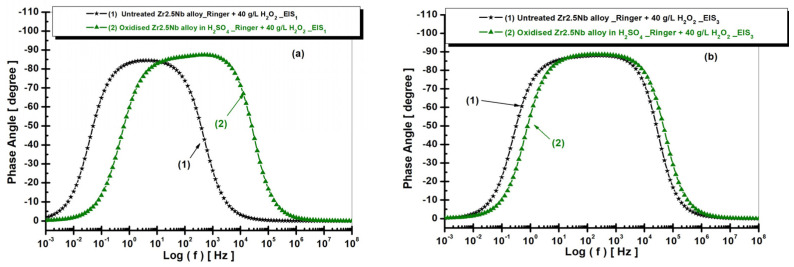
Comparative diagrams of electrochemical impedance spectroscopy (EIS) in Bode format with the representation of the phase angle as a function of the logarithm of frequency, for both untreated and electrochemically oxidized Zr2.5Nb samples after immersion in artificial Ringer’s solution with 40 g/L H_2_O_2_. Measurement performed in two time steps: (**a**) t_1_—5 h and (**b**) t_2_—77 h.

Figure 12 and Figure 13 present the EIS diagrams in Bode format with the representation of the impedance modulus as a function of the logarithm of frequency and with the representation of the phase angle as a function of the logarithm of frequency for both untreated and electrochemically oxidized Zr2.5Nb samples after immersion in Ringer’s solution with 40 g/L H_2_O_2_. The measurements are performed for the two studied times.

Analyzing the diagrams in Figure 12, it can be seen that the impedance of the Zr2.5Nb alloy immersed in Ringer’s solution with the addition of 40 g/L H_2_O_2_ is lower compared to the impedance obtained for the electrochemically oxidized sample.

From Figure 13, it is observed that the highest value of the phase angle, approximately −80°, is recorded for the alloy electrochemically oxidized in sulfuric acid solution (curve 2), which reflects an improved capacitive behavior and an optimal passivation compared to the other studied surfaces. The values of the equivalent circuit elements used for the simulation of the experimental data are presented in Table 2.

From Table 2, it can be observed that the evolution of the α parameter (EIS_1_/EIS_3_) for both the untreated and anodized Zr2.5Nb samples immersed in Ringer’s solution containing 40 g/L H_2_O_2_ reflects changes in the dielectric uniformity and stability of the passive films. For the untreated alloy, α values remain relatively low and almost constant (0.95), indicating a heterogeneous and defect-rich surface with a non-uniform oxide layer that shows limited protective capability under oxidative conditions.

In contrast, the anodized Zr2.5Nb sample initially exhibits higher α values (0.99), consistent with the formation of a compact and homogeneous anodic oxide layer. However, a slight decrease in α after prolonged immersion (EIS_3_) suggests that exposure to the strongly oxidative environment containing H_2_O_2_ induces partial modification or degradation of the oxide film. This decrease indicates a minor increase in surface heterogeneity, likely due to localized dissolution or structural rearrangement within the anodic oxide film.

Overall, although the anodized surface maintains a higher α compared to the untreated alloy, the observed reduction over time highlights the sensitivity of the passive film to aggressive oxidative conditions, suggesting that hydrogen peroxide can gradually affect its dielectric homogeneity and long-term stability.

The electrochemically oxidized sample exhibits higher R_p_ values compared to the untreated sample, indicating greater stability in the oxidative environment.

The passivation layer capacity decreases significantly for the samples oxidized in H_2_SO_4_, suggesting the formation of a denser and less porous protective layer compared to the untreated sample.

The electrochemically oxidized samples have significantly higher polarization resistance values than the untreated sample, suggesting improved corrosion protection.

These values suggest that electrochemical oxidation treatments in sulfuric acid solutions contribute to the formation of superior protective layers on the surface of the Zr2.5Nb alloy, thus optimizing its corrosion resistance in solutions similar to the physiological environment.

**Figure 14:**
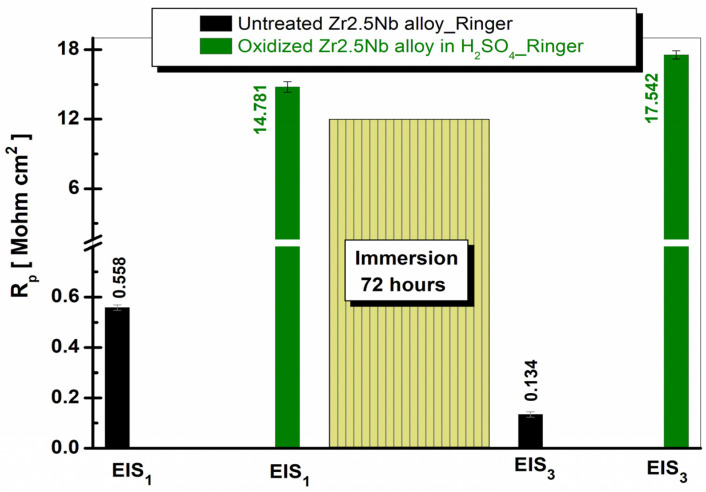
Comparative evolution of the polarization resistance (Rp) of the Zr2.5Nb alloy under study both in untreated form and anodically oxidized in sulfuric acid, for both electrochemical measurement times in artificial Ringer’s solution.

**Figure 15:**
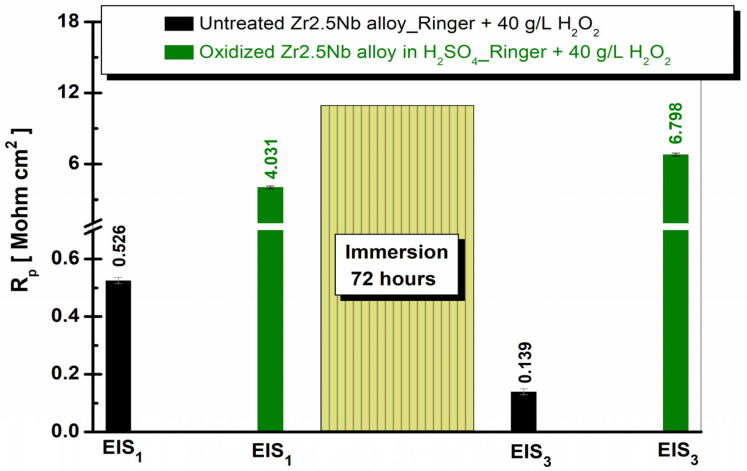
Comparative evolution of the polarization resistance (Rp) of the untreated and anodically oxidized Zr2.5Nb alloy in sulfuric acid, for both electrochemical measurement times in the solution composed of Ringer with 40 g/L H_2_O_2_.

To highlight the evolution of the polarization resistance (R_p_) obtained from EIS measurements for the Zr2.5Nb alloy, Figure 14 and Figure 15 present bar graphs, in which the R_p_ values are illustrated for the Zr2.5Nb alloys both in the untreated state and after anodic oxidation in sulfuric acid solution, immersed in Ringer’s solution and Ringer’s solution with 40 g/L H_2_O_2_ at both evaluated measurement times.

From Figure 14, we can see that the polarization resistance increases with the application of anodic oxidation treatment compared to the untreated sample. It is also observed that increasing the immersion time of the samples leads to an increase in R_p_ values.

From Figure 15, we can see that the physio-pathological environment composed of Ringer’s solution with 40 g/L H_2_O_2_ significantly influences all the surfaces studied.

Comparing Figure 14 and Figure 15, the most sensitive surfaces to Ringer’s solution with 40 g/L H_2_O_2_ proved to be in the case of electrochemically oxidized samples, but nevertheless they retained superior anti-corrosion properties to untreated samples.

In conclusion, the formation of a protective layer on the surface of a bio-alloy can reduce the corrosion rate of the implanted material in human pathophysiological environments frequently encountered post-operatively.

## 3. Materials and Methods

### 3.1. Zr2.5Nb Alloy

For this research, the Zr2.5Nb alloy is used, which is supplied by Evek GmbH, Mülheim an der Ruhr, Germany, in the form of a plate with initial dimensions of 500 mm × 500 mm × 1.5 mm. For the experiments, small plates cut to dimensions of 25 mm × 25 mm × 1.5 mm were used. The chemical composition of the Zr2.5Nb alloy is presented in Table 3, and the mechanical properties of the material are detailed in Table 4.

After cutting, a chemical degreasing step is applied with a 50 g/L NaOH solution, followed by a chemical pickling step in a dilute hydrochloric acid solution (HCl 1:1), both of which have the role of removing organic impurities and superficial oxidations. These treatments allow for the complete elimination of residual particles and traces of grease or contaminants from the surface of the samples. Next, the samples are rinsed with distilled water to eliminate traces of degreasers.

### 3.2. Electrochemical Oxidation Process

Electrochemical oxidation is performed using a TDK Lambda Gen300-8 (TDK-Lambda Corporation, Tokyo, Japan) electrochemical device in a classic electrochemical cell composed of two electrodes. Thus, the anode is the sample to be oxidized, and the cathode is a platinum–rhodium mesh, being inert in the oxidation solution. The cell also includes a cooling system to maintain the temperature of the acidic solution (200 mL of 1 M H_2_SO_4_) constant throughout the process. This configuration allows for precise control of the electrochemical oxidation process, favoring the formation of uniform nanostructures on the surface of the investigated alloy. Thus, once the circuit is closed, redox processes are initiated in the electrochemical anodization cell, using electrolytes such as sulfuric acid. The oxidation of the superficial layer, which functions as the anode, determines the release of metal cations, while reduction takes place at the cathode. Subsequently, the metal cations generated at the anode react with oxygen in the water, resulting in the formation of a uniform oxide layer on the metal surface. During the electrochemical oxidation process, an exposed surface area of 6.25 cm^2^ of the sample was used as the anode, while the remaining surface was insulated to ensure a uniform oxide growth over the active area.

For the subsequent corrosion measurements, the exposed area was reduced to 5 cm^2^ by additionally insulating part of the sample edges. This was done to minimize the influence of edge effects and surface irregularities, as the borders of the specimens tend to be rougher and may interfere with the accuracy and reproducibility of the electrochemical measurements.

Several experimental parameters were varied in the electrochemical oxidation process, including electrolytes, different values of the applied voltage (200 V, 250 V and 275 V) and different treatment times. As a result of the experiments, it was found that the most effective parameters were obtained in sulfuric acid solution, at a voltage of 200 V and an oxidation time of 1 min, conditions in which the most uniform oxide layers with the size of the pores formed in the nanometric range were formed. All electrochemical oxidation tests were performed at room temperature, 22 ± 1 °C, and were repeated 5 times to verify the reproducibility of the data.

### 3.3. Description of the Corrosion Test Method

The corrosion resistance of the samples is studied on a PGZ301 electrochemical apparatus with integrated VoltaMaster4 version 7.10 software, in an electrochemical cell composed of three electrodes (the working electrode is the surface to be studied, the working counter electrode (a Pt-Rh network) and a reference electrode (Ag/AgCl). The volume of electrolyte used was 150 mL simplified Ringer’s solution, and simplified Ringer’s solution with the addition of 40 g/L H_2_O_2_, which was used to simulate a highly oxidative environment associated with inflammation processes in the human body. The chemical composition and physico-chemical parameters of simplified Ringer’s solution are shown in Table 5. The characterization of the solutions in terms of pH, electrical conductivity and salinity is performed using a sensiIONTM + MM374 electrochemical multimeter (Hach, Loveland, CO, USA).

The electrochemical reagents used for the preparation of simplified Ringer’s solution were purchased from Sigma-Aldrich (Darmstadt, Germany). The 30% hydrogen peroxide used for the preparation of the electrolyte with properties similar to the inflammatory medium was also purchased from the same company.

In corrosion tests, the use of simplified Ringer’s solution and hydrogen peroxide (H_2_O_2_) is motivated by the need to simulate the real conditions of the human internal environment in which metallic implants are to be placed. Simplified Ringer’s solution, with an ionic composition close to that of blood plasma, is frequently used as a reference medium for evaluating the stability of materials under physiological conditions. It provides a buffered environment, with a neutral pH thus reflecting the normal state of the body, in the absence of inflammatory processes or other postoperative complications.

On the other hand, hydrogen peroxide (H_2_O_2_) is used to reproduce the inflammatory environment characteristic of the initial post-implantation stage. This is naturally generated by the body’s immune system, especially by leukocytes (neutrophils, macrophages), through the so-called oxidative burst, in which reactive oxygen species (ROS), including H_2_O_2_, are released. Thus, the addition of hydrogen peroxide to simplified Ringer’s solution allows for the investigation of the electrochemical behavior of the material in a pronounced oxidative environment, similar to that associated with inflammation and cellular oxidative stress.

Therefore, the choice of the two media, simplified Ringer’s solution and the solution with the addition of H_2_O_2_, offers a complex and relevant experimental approach, allowing for the evaluation of the corrosion resistance of the surfaces obtained both under normal physiological conditions and in the context of the challenges imposed by the pathological inflammatory environment.

The electrochemical experimental protocol applied for the study of electrochemical corrosion consisted of the application of two electrochemical methods: open circuit potential evolution (OCP) and electrochemical impedance spectroscopy (EIS) at a free potential, in alternating current with amplitude AC = 10 mV, at a variable frequency from 100 kHz to 10 mHz, where data recording is performed every 20 s. For fitting the EIS experimental data, Z_View_ 3.4 software is used, the chi-square value obtained from fitting the experimental data being less than 10^−3^.

The experiments were carried out at a controlled temperature of 37 ± 1.5 °C, corresponding to the physiological temperature of the human body, using a thermostated system to maintain constant solution temperature during the tests.

### 3.4. Morphological and Compositional Characterization (SEM–EDX)

The surface morphological and compositional analysis of the Zr2.5Nb alloy and the obtained oxide films were performed using the FEI QUANTA 200 SEM (FEI Company, Hillsboro, OH, USA) scanning electron microscope (SEM) and the energy dispersive X-ray analyzer (EDX) coupled to the EDAX Genesis data acquisition 5.10 software.

### 3.5. Structural Analysis with X-Ray Diffractometer

X-ray diffraction (XRD) measurements were performed on a Dron-3 equipment using Co Kα (λ = 1.790300 Å). The X-ray diffractometer was operated at a voltage of 30 kV and a current of 20 mA, with a step of 0.05°/s, an exposure of 3 s and a total time/sample of 2 h and 13 min, in a range of 2θ = 15–90°. The obtained diffraction patterns were analyzed using the Match! 3.16 software, and the reference database library used was the Crystallography Open Database (COD).

## 4. Conclusions

The results of this study demonstrate the superiority of the electrochemical oxidation treatment compared to the untreated Zr2.5Nb alloy in forming oxide layers with enhanced anti-corrosion properties. The treatment performed in 1 M H_2_SO_4_ at 200 V for 1 min proved to be the most effective condition for obtaining a homogeneous oxide film with nanometric porosity, which significantly improves the protective behavior of the alloy in simulated physiological and inflammatory environments.

SEM–EDX analysis revealed that electrochemical oxidation under these conditions promotes the formation of uniformly distributed nanopores and leads to a noticeable increase in oxygen content, confirming effective surface oxidation compared to the untreated sample. XRD analysis indicated that electrochemical oxidation induces a structural transformation from the monoclinic baddeleyite (ZrO_2_) phase, characteristic of the untreated alloy, to the more stable tetragonal arkelite (ZrO_2_) phase. This transformation contributes to the improved corrosion resistance of the treated material through the formation of a denser and more stable oxide layer.

Electrochemical investigations confirmed that anodically oxidized samples exhibit more positive open circuit potential values and significantly higher polarization resistance compared to untreated ones. These findings demonstrate that the electrochemical oxidation process produces a compact and adherent oxide layer that effectively limits electrochemical reactions at the metal–electrolyte interface.

Even though the addition of hydrogen peroxide (H_2_O_2_) simulating an inflammatory environment negatively affected the overall corrosion behavior, the oxidized samples maintained superior stability and higher resistance compared to untreated ones. This observation suggests that the oxide film generated by the electrochemical oxidation process offers long term protection, even under oxidative stress conditions.

In conclusion, anodic oxidation in sulfuric acid at 200 V for 1 min represents an optimal surface treatment for biomedical applications, promoting the development of durable, corrosion-resistant oxide layers that can extend implant longevity under physiological and oxidative conditions relevant to the in vivo environment.

## Figures and Tables

**Table 1:** The determined values of the representative equivalent electrical circuit for each type of surface analyzed, after experimental fitting of Zr2.5Nb samples, both untreated and electrochemically oxidized in 1 M H_2_SO_4_, after immersion in Ringer’s solution.

Equivalent Electrical Circuit Elements/Measuring Unit	Untreated Zr2.5Nb EIS_1_/EIS_3_	Oxidized Zr2.5Nb Eox 200 V–1 min 1 M H_2_SO_4_ EIS_1_/EIS_3_
**Rs** [Ω cm^2^]	46.92 / 48.59	66.53 / 59.37
**CPE-P** [F/cm^2^]	6.999 × 10^−6^ / 5.887 × 10^−6^	6.422 × 10^−8^ / 2.296 × 10^−9^
**α**	0.920 / 0.924	0.977 / 0.971
**Rp** [MΩ cm^2^]	0.5581 / 0.1342	16.82 × 10^−6^ / 3.375 × 10^−6^
**CPE-Tox** [F/cm^2^]	-	1.447 × 10^−9^ / 6.411 × 10^−8^
**α**	-	0.831 / 0.976
**Rp** [MΩ cm^2^]	-	14.7810 / 17.5420

**Table 2:** The determined values of the representative equivalent electrical circuit for each type of surface analyzed, after experimental fitting of Zr2.5Nb samples, both untreated and electrochemically oxidized in 1 M H_2_SO_4_, after immersion in the solution composed of Ringer with 40 g/L H_2_O_2_.

Equivalent Electrical Circuit Elements/Measuring Unit	Untreated Zr2.5Nb EIS_1/_EIS_3_	Oxidized Zr2.5Nb Eox 200 V–1 min 1 M H_2_SO_4_ EIS_1_/EIS_3_
**Rs** [Ω cm^2^]	62.22 / 60.9	87.79 / 67.70
**CPE-P** [F/cm^2^]	7.466 × 10^−6^ / 6.451 × 10^−6^	6.189 × 10^−8^ / 7.984 × 10^−8^
**α**	0.952 / 0.952	0.998 / 0.998
**Rp** [MΩ cm^2^]	0.5257 / 0.1397	0.14625 / 0.02361
**CPE-Tox** [F/cm^2^]	-	3.366 × 10^−8^ / 6.943 × 10^−8^
**α**	-	0.735 / 0.450
**Rp** [MΩ cm^2^]	-	4.0304 / 6.7985

**Table 3:** Chemical composition of Zr2.5Nb alloy [wt.%].

Be	Hf	Ni	Cr	Ti	Al	O	Pb	Nb	Zr
0.003	0.01	0.02	0.02	0.007	0.008	0.06–0.1	0.005	2.4–2.7	Rest

**Table 4:** Mechanical properties at 20 °C of the Zr2.5Nb alloy.

Alloy	Modulus of Elasticity (Young) [GPa]	Breaking Strength [MPa]	Elongation δ [%]	Hardness HB [kgf/mm^2^]
Zr2.5Nb	95	569	28	64–67

**Table 5:** Chemical composition and physico-chemical parameters of simplified Ringer’s solution and simplified Ringer’s solution with inflammatory compound.

Nr. Crt.	Chemical Compound	Solution Name Simplified Ringer [g/L]	Simplified Ringer + 40 g/L H_2_O_2_
1	NaCl	8.402	8.402
2	KCl	0.302	0.302
3	CaCl_2_	0.298	0.298
4	H_2_O (distilled water)	Rest	Rest
5	pH	6.67	5.85
6	Conductivity [mS/cm]	14.4	12.3
7	Salinity [ppt]	8.4	7.1

## Data Availability

The original contributions presented in this study are included in the article. Further inquiries can be directed to the corresponding authors.

## References

[1] Erwin A.V. (2013). Surface Modification for Biocompatibility. Engineered Biomimicry.

[2] Abd El-Ghany O.S., Sherief A.H. (2016). Zirconia based ceramics, some clinical and biological aspects: Review. Future Dent. J..

[3] Nie L., Zhan Y., Liu H., Tang C. (2014). Novel β-type Zr–Mo–Ti alloys for biological hard tissue replacements. Mater. Des..

[4] Li C., Zhan Y., Jiang W. (2011). Zr–Si biomaterials with high strength and low elastic modulus. Mater. Des..

[5] Hua N., Huang L., Wang J., Cao Y., He W., Pang S., Zhang T. (2012). Corrosion behavior and in vitro biocompatibility of Zr–Al–Co–Ag bulk metallic glasses: An experimental case study. J. Non-Cryst. Solids.

[6] Lu X., Huang L., Pang S., Zhang T. (2012). Formation and biocorrosion behavior of Zr-Al-Co-Nb bulk metallic glasses. Chin. Sci. Bull..

[7] Mehjabeen A., Song T., Xu W., Tang H.P., Qian M. (2018). Zirconium alloys for orthopaedic and dental applications. Adv. Eng. Mater..

[8] Eliaz N. (2019). Corrosion of Metallic Biomaterials: A Review. Materials.

[9] Li H.-Z., Zhao X., Xu J. (2015). MRI-compatible Nb–60Ta–2Zr alloy for vascular stents: Electrochemical corrosion behavior in simulated plasma solution. Mater. Sci. Eng. C Mater. Biol. Appl..

[10] Virtanen S., Milošev I., Gomez-Barrena E., Trebše R., Salo J., Konttinen Y.T. (2008). Special modes of corrosion under physiological and simulated physiological conditions. Acta Biomater..

[11] Nadaraia K.V., Mashtalyar D.V., Piatkova M.A., Pleshkova A.I., Imshinetskiy I.M., Gerasimenko M.S., Belov E.A., Kumeiko V.V., Kozyrev D.N., Fomenko K.A. (2024). Antibacterial HA-coatings on bioresorbable Mg alloy. J. Magnes. Alloys.

[12] Gilbert J.L., Mali S.A., Eliaz N. (2012). Medical Implant Corrosion: Electrochemistry at Metallic Biomaterial Surfaces. Degradation of Implant Materials.

[13] Shilong L., Lei S., Yingjie G., Jingyang W., Di L., Shenlong Z. (2024). Selective oxygen reduction reaction: Mechanism understanding, catalyst design and practical application. Chem. Sci..

[14] Katunar M.R., Gomez Sanchez A., Santos Coquillat A., Civantos A., Martinez Campos E., Ballarre J., Vico T., Baca M., Ramos V., Cere S. (2017). In vitro and in vivo characterization of anodised zirconium as a potential material for biomedical applications. Mater. Sci. Eng. C Mater. Biol. Appl..

[15] Sanchez A.G., Ballarre J., Orellano J.C., Duffó G., Cere S. (2013). Surface modification of zirconium by anodisation as material for permanent implants: In vitro and in vivo study. J. Mater. Sci. Mater. Med..

[16] Mousavi-Semnani S.Z., Yousefpour M., Zareidoost A. (2022). Enhancing the biocompatibility of ZrO_2_ thin film on Zr-2.5Nb alloy by anodizing treatment using an electrolyte containing biofunctional groups. Thin Solid Films.

[17] Song K.Y., Zhang H., Zhang W.J., Teixeira A. (2018). Enhancement of the surface free energy of PDMS for reversible and leakage-free bonding of PDMS–PS microfluidic cell-culture systems. Microfluid. Nanofluidics.

[18] bin Anwar Fadzil A.F., Pramanik A., Basak A.K., Prakash C., Shankar S. (2022). Role of surface quality on biocompatibility of implants—A review. Ann. 3D Print. Med..

[19] Pralhad P., Shivprakash B. (2021). Surface modification of titanium and titanium alloy by plasmaelectrolytic oxidation process for biomedical applications: A review. Mater. Today Proc..

[20] Mori Y., Masahashi N., Aizawa T. (2022). A Review of anodized TiNbSn alloys for improvement in layer quality and application to orthopedic implants. Materials.

[21] Lestari F.P., Sari Y.R., Rokhmanto F., Asmaria T., Pramono A.W. (2020). Surface modification of Ti-6Al-4V alloy by anodization technique at low potential to produce oxide layer. J. Electron. Electromed. Eng. Med. Inform..

[22] Benea L., Ravoiu A., Neaga V., Axente E.R. (2023). Using Applied Electrochemistry to Obtain Nanoporous TiO_2_ Films on Ti_6_Al_4_V Implant Alloys and Their Preclinical In Vitro Characterization in Biological Solutions. Coatings.

[23] Gomez Sanchez A., Katunar M., Ceré S., Grzegorz D.S. (2020). Chapter ten—Structural characteristics and barrier properties of anodic zirconium oxides for biomedical applications. Micro and Nano Technologies Nanostructured Anodic Metal Oxides.

[24] Schünemann F.H., Galárraga-Vinueza M.E., Magini R., Fredel M., Silva F., Souza J.C.M., Zhang Y., Henriques B. (2019). Zirconia surface modifications for implant dentistry. Mater. Sci. Eng. C Mater. Biol. Appl..

[25] Zhou F.Y., Qiu K.J., Bian D., Zheng Y.F., Lin J.P. (2014). A Comparative in vitro study on biomedical Zr-2.5X (X = Nb, Sn) alloys. J. Mater. Sci. Technol..

[26] Romonti D.E., Gomez Sanchez A.V., Milošev I., Demetrescu I., Ceré S. (2016). Effect of anodization on the surface characteristics and electrochemical behaviour of zirconium in artificial saliva. Mater. Sci. Eng. C.

[27] Cox B., Gascoin F., Wong Y.M. (1995). Properties of thin anodic oxide films on zirconium alloys. J. Nucl. Mater..

[28] Epp J., Hübschen G., Altpeter I., Tschuncky R., Herrmann H.G. (2016). 4—X-ray diffraction (XRD) techniques for materials characterization. Materials Characterization Using Nondestructive Evaluation (NDE) Methods.

[29] Khan H., Yerramilli A.S., D’Oliveira A., Alford T.L., Boffito D.C., Patience G.S. (2020). Experimental methods in chemical engineering: X-ray diffraction spectroscopy—XRD. Can. J. Chem. Eng..

[30] Motta A., Yilmazbayham A., Comstock R., Partezana J., Sabol G.P., Lai B., Cai Z. (2005). Microstructure and Growth Mechanism of Oxide Layers Formed on Zr Alloys Studied with Micro-Beam Synchrotron Radiation. J. ASTM Int..

[31] Silva C., Leonard K., Trammel M., Bryan C. (2018). Characterization of different forms of Zr-2.5Nb samples before and after neutron irradiation. Mater. Sci. Eng. A.

[32] Mangla O., Roy S. (2019). Monoclinic Zirconium Oxide Nanostructures Having Tunable Band Gap Synthesized under Extremely Non-Equilibrium Plasma Conditions. Proceedings.

[33] Mordyuk B.N., Karasevskaya O.P., Prokopenko G.I. (2013). Structurally induced enhancement in corrosion resistance of Zr–2.5%Nb alloy in saline solution by applying ultrasonic impact peening. Mater. Sci. Eng. A.

[34] Straumal B., Gornakova A., Fabrichnaya O., Kriegel M., Mazilkin A., Baretzky B., Gusak A., Dobatkin S. (2012). Effective temperature of high pressure torsion in Zr-Nb alloys. High Temp. Mater. Process..

[35] Branzoi I.V., Iordoc M., Codescu M. (2008). Electrochemical studies on the stability and corrosion resistance of new zirconium-based alloys for biomedical applications. Surf. Interface Anal..

[36] Praveen P., Viruthagiri G., Mugundan S., Shanmugam N. (2014). Structural, optical and morphological analyses of pristine titanium di-oxide nanoparticles—Synthesized via sol–gel route. Spectrochim. Acta Part A Mol. Biomol. Spectrosc..

[37] Benea L., Simionescu-Bogatu N. (2021). Reactivity and Corrosion Behaviors of Ti_6_Al_4_V Alloy Implant Biomaterial under Metabolic Perturbation Conditions in Physiological Solutions. Materials.

[38] Benea L., Mardare-Danaila E., Mardare M., Celis J.P. (2014). Preparation of titanium oxide and hydroxyapatite on Ti–6Al–4V alloy surface and electrochemical behaviour in bio-simulated fluid solution. Corros. Sci..

[39] Benea L., Simionescu N. (2021). Impact of hydrogen peroxide and albumin on the corrosion behavior of titanium alloy (Ti_6_Al_4_V) in saline solution. Int. J. Electrochem. Sci..

[40] Zhou F.Y., Wang B.L., Qiu K.J., Lin W.J., Li L., Wang Y.B., Nie F.L., Zheng Y.F. (2012). Microstructure, corrosion behavior and cytotoxicity of Zr–Nb alloys for biomedical application. Mater. Sci. Eng. C.

[41] Sowa M., Dercz G., Suchanek K., Simka W. (2015). Investigation of anodic oxide coatings on zirconium after heat treatment. Appl. Surf. Sci..

[42] Sowa M., Łastówka D., Kukharenko A.I., Korotin D.M., Kurmaev E.Z., Cholakh S.O., Simka W. (2017). Characterisation of anodic oxide films on zirconium formed in sulphuric acid: XPS and corrosion resistance investigations. J. Solid State Electrochem..

[43] Neaga V., Benea L., Axente E.R. (2022). Corrosion Assessment of Zr2.5Nb Alloy in Ringer’s Solution by Electrochemical Methods. Appl. Sci..

[44] Benea L., Ravoiu Lupu A., Bounegru I., Vizureanu P. (2023). Effect of functional nanoporous TiO_2_ film obtained on Ti_6_Al_4_V implant alloy to improve resistance in biological solution for inflammatory conditions. Int. J. Mol. Sci..

[45] Zhang Y., Addison O., Yu F., Troconis B.C.R., Scully J.R., Davenport A.J. (2018). Time-dependent enhanced corrosion of Ti_6_Al_4_V in the presence of H_2_O_2_ and albumin. Sci. Rep..

[46] Ravoiu A. (2023). Effect of Surface Modification of Ti_6_Al_4_V Alloys on Their Behavior in the Biological Implant Environment Under Inflammatory Conditions. PhD Thesis.

[47] Uttiya S., Contarino D., Prandi S., Carnasciali M.M., Gemme G., Mattera L., Rolandi R., Canepa M., Cavalleri O. (2014). Anodic oxidation of titanium in sulphuric acid and phosphoric acid electrolytes. J. Mater. Sci. Nanotechnol..

[48] Trentin A., Pakseresht A., Duran A., Castro Y., Galusek D. (2022). Electrochemical Characterization of Polymeric Coatings for Corrosion Protection: A Review of Advances and Perspectives. Polymers.

[49] Nkomo D., Masia N., Fahmina Z., Anujit G., Eram S. (2021). An insight on corrosion resistance ability of biocompatible dental implants through electrochemical impedance spectroscopy. Corrosion—Fundamentals and Protection Mechanisms.

[50] Okazaki Y., Nagata H. (2012). Comparisons of immersion and electrochemical properties of highly biocompatible Ti–15Zr–4Nb–4Ta alloy and other implantable metals for orthopedic implants. Sci. Technol. Adv. Mater..

